# Do we cope similarly with different adversities? COVID-19 versus armed conflict

**DOI:** 10.1186/s12889-022-14572-0

**Published:** 2022-11-23

**Authors:** Shaul Kimhi, Hadas Marciano, Yohanan Eshel, Bruria Adini

**Affiliations:** 1 ResWell Research Collaboration, Tel Aviv University, Tel Aviv, Israel; 2 Stress and Resilience Research Center, Tel-Hai College, Tel–Hai, Israel; 3 The Institute of Information Processing and Decision Making, University of Haifa, Haifa, Israel; 4 The Psychology Department, University of Haifa, Haifa, Israel; 5 Department of Emergency and Disaster Management, School of Public Health, Sackler Faculty of Medicine, Tel Aviv University, Tel Aviv, Israel

**Keywords:** COVID-19, Armed conflict, Distress, Resilience, Wellbeing

## Abstract

**Background:**

Varied populations may react differently to similar crises, depending on their social, cultural, and personal backgrounds; conversely, the same populations may respond differently to varied adversities. The current study aimed to examine three types of resilience (individual, community, and societal resilience) predicting six coping mechanisms (sense of danger, anxiety and depressive symptoms, well-being, hope, and morale) among the same sample of people that faced across two different adversities—COVID-19 and an armed conflict.

**Methods:**

Two repeated measurements of the same Israeli sample (*N* = 593) were employed, through an internet panel. The research variables were examined through a structured, quantitative questionnaire that consisted of nine scales, based on validated and reliable questionnaires.

**Results:**

Results indicated that: (a) respondents reported more difficulties in coping with the COVID-19 crisis, compared to the armed conflict, in all variables but morale. (b) similar patterns of correlations among the study variables were found in both measurements. (c) path's analysis indicated similar patterns of prediction of distress and well-being by individual and societal resilience. Use of the coping mechanism varied depending on the perception of the threat: COVID -19 is perceived as a less familiar and predictable adversity, which is harder to cope with, compared with the more familiar risk – an armed conflict, which is a recurrent threat in Israel. The correlations between the investigated psychological responses and the impacts of resilience on the coping and distress mechanism were similar in both adversities.

**Conclusions:**

The results indicate that respondents tend to react in a similar pattern of associations among resilience, distress, and well-being across different adversities, such as COVID and armed conflict. However, individuals tend to regard unfamiliar, less predictable adversities as more complex to cope with, compared to better-known crises. Furthermore, respondents tend to underestimate the risks of potential familiar adversities. Healthcare professionals must be aware of and understand the coping mechanisms of individuals during adversities, to appropriately design policies for the provision of medical and psychological care during varied emergencies.

## Introduction

Populations worldwide need to manage varied types of hazards resulting from different adversities, such as natural disasters, pandemics, and/or human-made events. Adversities impact the resilience and well-being of individuals and communities, subsequently followed by elevated levels of distress symptoms [1]. Varied populations may react differently to similar crises, dependent on their social, cultural, and personal backgrounds and experiences [2, 3]. Furthermore, the same population may respond differently to varied adversities, presenting different levels of distress, resilience, and well-being in each event. At times, different types of adversities may occur simultaneously, for example, hurricanes, flooding, earthquakes, armed conflicts, etc. that occurred in numerous countries, in parallel to the ongoing global COVID-19 pandemic [4, 5]. It is thus of value to identify similarities and differences in coping with varied adversities.

### Two types of adversities that occurred concurrently

The COVID-19 pandemic started in Israel upon the initial identification of confirmed cases in February 2020. It continued in three main waves and substantially receded at the beginning of 2021, following a successful vaccination campaign. By April 19^th^, 2021, 88% of individuals the age of 50 years or higher were vaccinated with two vaccine doses [6]. By June 21 Israel faced the fourth wave of morbidity which led to a decision to inoculate the adult population with a third (booster) vaccination. As of February 7^th^, 2022, Israel was at the peak of the fifth wave resulting from the Omicron variant, leading to the overall accumulated deaths resulting from COVID of 8,272 Israelis.

Concurrently, an armed conflict between Israel and the Palestinians in Gaza Strip erupted, starting on May 10, 2021, and ending in a ceasefire that was declared 11 days later, on May 21, 2021. Ten civilians and one Israeli soldier were killed. During the data collection process that was conducted during the armed conflict, the coronavirus was in a significant decline in Israel (https://www.worldometers.info/coronavirus/country/israel). For example, the number of new cases discovered during the operation was about 30 people per day, while during the third wave that preceded this period (December 2020 to March 2021) there were thousands of new cases per day (://coronavirus.jhu.edu/region/israel).

The two adversities that our respondents coped with posed different challenges: The COVID-19 pandemic was characterized by great uncertainty, especially before the development of a vaccine, affecting many aspects of life [7]. Among the varied uncertainties, the following should be noted: a high death toll was witnessed due to the pandemic, it was an emerging threat, unfamiliar to most people, the virus was invisible and concerning, it was unclear when a vaccine will be available, carriers that did not show symptoms could not be identified, and more [8]. Furthermore, this was the first pandemic with massive consequences that negatively impacted the daily lives of most people worldwide by requiring social distancing, isolation, and national lockdowns [9].

The period of an armed conflict (Operation Guardian of the Walls) that occurred approximately seven months after the peak of the COVID-19 pandemic in Israel, posed a direct threat to the lives of civilians in large areas of Israel [10, 11]. Residents of many communities were instructed to remain in or near sheltered infrastructures throughout the conflict. Based on previous experience with such rounds of conflicts, most residents had a high level of trust in the missile defense system (Iron Dome) [12], believing that it will protect them by intercepting most of the rockets aimed at their area. They also assumed, based on the former conflicts, that the hostilities will be relatively short, and that the various authorities (military, hospitals, municipalities) will come to their aid immediately if needed. During the military conflict, the various media in Israel dealt solely, and on a daily basis, with this round of fighting (e.g., https://www.kan.org.il/tags/tag.aspx?tagid=2426).

The current study aimed to examine whether there are differences in coping with these two different types of adversities: the COVID-19 pandemic (global adversity resulting from a natural cause) versus a human-made armed conflict. The levels of distress, resilience, and well-being were assessed among the same sample of the population to examine commonalities and differences in dealing with the two different crises, that occurred within a relatively short period. To the best of our knowledge, to date, these issues have hardly been explored as it is infrequent that two varied adversities materialize concurrently.

### Distress symptoms

In 1950, Selye argued that “anything that causes stress endangers life unless it is met by adequate adaptive responses; conversely, anything that endangers life causes stress and adaptive responses” [13], p. 1383]. Since this pioneered publication the concept of distress-related health implications has been widely studied (see comprehensive review in [14]). Distress is a broad concept that refers to a wide range of individual reactions in response to environmental demands, such as various threats. Of the potential mechanism of distress, two were frequently investigated: (a) Distress symptoms, which form a common reaction to crises characterized by physical or mental threats to the integrity of life. Such symptoms comprise continuous emotional and behavioral difficulties [15] including depression, anxiety, and grief [16]. Anxiety and depression symptoms were identified as a common response to both COVID-19 [17] and armed conflicts [18]. (b) A sense of danger is likely to enhance a continuous state of fear that strongly and negatively reduces the capacity to effectively cope with adversities [19].

### Resilience

The concept of resilience has received a great deal of research attention in the past decades, and even more so in the last few years. Presumably, this is related to the onset of several pandemics as well as the rising climate change crises and disasters that follow [4]. Despite the numerous definitions of resilience that can be found in the literature, there is a broad consensus that human resilience mainly refers to the human ability to cope with crises successfully and effectively recover as quickly as possible [20].

The study focused on three main types of resilience, as follows: (a) Individual resilience (IR), which relates to the capacity of the individual to endure adversities, recuperate and bounce back (or forward) to routine function [21]. Cacioppo et al., [22] described the concept of individual resilience as “the capacity to foster, engage in, and sustain positive relationships and to endure and recover from life stressors and social isolation” (p. 44). (b) Community resilience (CR), relates to the relationships between the people and their community, mainly focusing on the effectiveness of the community to successfully attend to the specific requirements of its population and the degree to which people receive assistance from their neighbors, peers, and entities on which their community consists of [23]. (c) National resilience (NR), is a comprehensive concept that focuses mainly on social sustainability and empowerment concerning the following components: confidence (trust) in the government, the parliament, and other societal entities, faith in social cohesion, and patriotism [24]. Previous studies [25] have revealed that the three resilience measurement tools (regarding IR, CR, and SR) positively predicted both hope and morale and negatively predicted distress during COVID-19.

### Well-being

The concept of well-being (WB) is complex, characterized by many definitions, and varied in how it is measured (see an extensive review in [26]. Beyond the various definitions, this concept includes several aspects such as positive versus negative affect, life satisfaction, relationships with others, purpose in life, happiness, and more. Above all, it is a measurement of the individual's subjective assessment of his/her life. Earlier studies have examined well-being during COVID-19 [27] as well as during armed conflicts [28].

### Morale

Shaban et al., [29] defined morale as "a quality which involves feelings, emotions, attitude, and perception towards the organization and its members". The concept of morale emerged in the military context (also known as 'esprit de corps, [30]). Morale is defined in terms of the mental, spiritual, emotional, and general state of the individual, rather than in terms of a personality attribute [31].

### Hope

Snyder et al., [32] emphasized the cognitive aspect of hope and defined it as a positive motivational state that is based on an interactively derived sense of successful (a) agency (goal-directed energy) and (b) pathways (planning to meet goals). Fredrickson [33] referred to hope mainly as an emotional aspect. Research has indicated a significant association between hope and stressful life events [34]. Gallagher et a., [35] suggest that hope is positively associated with resilience that enables one to successfully cope with the chronic stressors that are caused by the COVID-19 pandemic.

### Risk perception

The risk perception theory [36] claims that personal experience and social factors, along with connection with close environments, may exert significant effects on risk perception about adversities. Armed conflict crises have occurred many times in Israel and the population is thus familiar with the characteristics of these adversities including how they are expected to behave throughout these incidents. The COVID-19 pandemic, which is unexpectedly enhanced and decreased throughout the varied waves of infectivity, is a new experience for the Israeli public. Accordingly, the population lacks the means for assessing if, when, and how it will end, or what side effects will materialize. It is expected, therefore, that coping with this (previously unknown) pandemic will be regarded by the investigated public as a more complex task than coping with a (familiar) struggle with the Gaza Strip.

Moreover, the scientific relevance of the current study is related, among others, to the fact that different disasters may occur simultaneously (e.g., a severe wave of heat and drought and at the same time a pandemic crisis) and affect how one copes with the varied situations. Hence the importance of this study is the capacity to follow the same people, concerning their coping with two adversities, in a relatively short period. To date, it seems that this issue has not received extensive research coverage.

### Research hypotheses

Based on the above the following hypotheses were examined:

- The level of anxiety and depressive symptoms, as well as the sense of danger will be higher during the COVID-19 pandemic, compared with their level during the armed conflict. .
- The levels of resilience (IR, CR, and SR), Well-being, morale, and hope will be lower during the COVID-19 pandemic, compared with their level during the armed conflict.
- The three types of resilience will positively predict the coping mechanism (WB, morale, and hope), and negatively predict the markers of distress (anxiety and depressive symptoms, and sense of danger) in each of the two investigated adversities.

## Method

### Study design

The present study is longitudinal research, based on two repeated measurements that were conducted among the same sample. The first measurement (T1) was carried out at the peak of the COVID-19 pandemic in mid-October 2020 (October 12–14) towards the end of the second lockdown, before the beginning of the vaccination campaign. The second measurement (T2) took place between 14–16 May 2021, at the end of the armed conflict between Israel and the Gaza Strip. A previous study explored the associations between morale and perceived threats to the Israeli population in each of these two adversities, using two different cohorts of respondents [37].

### Participants

To collect our data, we used an internet panel company that consists of over 65,000 panelists, representing all demographic sectors and geographic locations in Israel (https://sekernet.co.il/). Our sample included 593 Jewish Israeli respondents, who answered the online questionnaire twice. An informed consent form was signed by all participants. The questionnaire was approved by the Ethics Committee of Tel Aviv University. All methods were carried out following relevant guidelines and regulations. The sociodemographic characteristics of the sample are detailed in Table 1.

**Table 1:** Sociodemographic characteristics of participants (*N* = 593)

Variable	Groups	N	%	M	S.D
Age groups	1. 18–30	83	14	47.74	15.22
	2. 31–40	131	22		
	3. 41–50	128	22		
	4. 51–60	108	18		
	5. 61—on	143	24		
Gender	1. Women	270	45		
	2. Men	323	55		
Income level	1. Much below	174	29	2.50	1.27
	2. Below	133	22		
	3. Average	140	24		
	4. Above	105	18		
	5. Much above	41	7		
Political attitudes	1. Much left	7	1	3.47	.85
	2. Left	65	11		
	3. Center	218	37		
	4. Right	247	42		
	5. Much right	56	9		
Religiosity	1. Secular	306	52	1.77	.95
	2. Traditional	161	27		
	3. Religious	81	14		
	4. Orthodox	45	7		
Education	1. Elementary	2	.3	3.37	1.09
	2. High School	132	22		
	3. Higher education	203	34		
	4. Academic B.A	156	26		
	5. Academic M.A. and above	100	17		

The answers concerning income level (about the average income of a family in Israel) and political attitudes represent the self-classifications of the respondents

*M*  Mean, *S.D*  Standard deviation

### Study tool

The research variables were examined with a structured, quantitative questionnaire that consisted of the following eight categories, based on validated and reliable questionnaires. The following are details of the same scales (research variables) in each of the measurements (T1 = COVID-19 and T2 = armed conflict).

### Sense of danger

The initial six-item Sense of Danger Scale was used based on Solomon and Prager's [38] scale, to measure a lingering sense of danger in the context of security threats. In our COVID-19 studies [18], we modified the threat in each of the items from security to the COVID-19 pandemic threat (e.g., “To what extent are you worried about the increase of the COVID-19 global crisis?"). To the six items' original scale we added four items, for example, "To what extent are you afraid that you will have difficulty finding work after the coronavirus crisis?" In the armed conflict questionnaire, the original “security” threat was measured. These ten items were rated on a scale ranging from 1 = Not at all, to 5 = Very much. The scale's Cronbach's alpha reliability in the current study was α = 0.88 in both measurements).

### Distress symptoms

The level of individual distress symptoms, in the context of the two adversities, was determined by nine items from the Brief Symptom Inventory (BSI, [39] regarding anxiety (four items) and depression (five items). This scale was scored on a Likert scale ranging from 1 = not suffering at all to 5 = suffering very much. An example of an item is: "Lack of interest in anything" The Cronbach alpha reliabilities of this scale in the present study were high: α = 0.91 at T1 and T2.

### Individual resilience (IR)

The original Connor-Davidson scale consists of 25 items [40]. In this study, we used an abbreviated version of the questionnaire, based on ten items [41]. Responses to the questionnaire items represent a 5-point scale, ranging from 1 = not true at all, to 5 = true almost all the time. Example of an item: “I can adapt when changes occur.” In the present study, the internal reliability of the scale was high: α = 0.91 (T1 and T2).

### Community resilience (CR)

This resilience scale includes 10 items [42]. Responses to the questionnaire items represent a 5-point scale, ranging from 1 = do not agree at all, to 5 = agree to a very large extent. Example of an item: "The municipal authority in my locality is functioning properly in the crisis". The current study's internal scale reliabilities were high (α = 0.94 at T1 and T2).

### Societal (national) resilience (SR)

The scale includes 16 items [43]. Example of an item: "I have full confidence in the ability of the Israeli healthcare system to take care of the population during the crisis". The response scale for the national resilience items ranges from 1 = do not agree at all to 6 = strongly agree. The internal reliability of the scale was high: α = 0.90 (T1) and α = 0.91 (T2).

### Subjective well-being

This scale consists of nine items concerning individuals' perceptions of their lives in the present regarding various contexts, such as work, family life, health, free time, and others [44]. Responses to these items range from 1 = very bad to 6 = very good. Example of an item: "How is your health today?". The reliabilities in the current study were found to be good in both measurements (α = 0.88 at T1 and α = 0.90 at T2).

### Morale

The level of personal morale was examined by a single item: “How would you define your morale these days?” The response scale ranges from 1 = not good at all, to 5 = very good.

### Level of hope

This tool, which was constructed specifically for the present study, is based on an earlier scale [45]. The scale contains two dimensions: personal and collective hope. The scale includes five items. Two of them refer to the personal level (e.g., "I hope that I will emerge strengthened from the coronavirus crisis") and three items refer to the collective level (e.g., "I hope that Israeli society will emerge strengthened from the coronavirus crisis"). The response scale ranges from 1 = very little hope to 5 = high hope. The internal reliability of the scale in the present study was found to be high: α = 0.93 (T1 and T2).

### Demographic characteristics

The demographic characteristics included: age group, gender, income relative to the average income in Israel (1 = much below the average, 5 = much above the average), political positions (1 = strong left, 5 = strong right), degree of religiosity (1 = secular, 4 = very religious/orthodox), education (1 = elementary school, 5 = academic master's degree).

### Statistical analysis

Three analysis methods were employed: (a) General linear Model repeated measures examined differences between the coping mechanisms across the two adversities. (b) Pearson correlations among the research variables examined the pattern of associations among the coping mechanisms, across the two adversities. (c) Two path analyses examined the prediction of six coping mechanisms, by the three types of resilience, across the two adversities. For the repeated measures, the mean scores of each of the scales were used. *P*-values lower than 0.05 were considered statistically significant.

## Results

To examine our first hypothesis, regarding the differences in coping mechanisms, we used General Linear Model (GLM) repeated measures (Table 2). Results indicated that in agreement with our hypothesis, respondents reported more difficulties in coping with the COVID-19 crisis, compared to the armed conflict, in all nine variables except for morale. They reported a higher level of stress symptoms during this pandemic (F = 7 0.84, *p* < 0.05), and a higher level of sense of danger, with the largest difference between the two adversities (F = 196.11, *p* < 0.001, with a medium-size effect). Respondents reported significantly higher levels of individual (F = 5.65, *p* < 0.01) (small effect size), community (F = 63.54, *p* < 0.001, small effect size), and societal (F = 60.41, *p* < 0.001, large effect size) resilience during the armed conflict measurement, compared to the COVID-19 measurement. Furthermore, higher levels of subjective well-being (F = 110.26, *p* < 0.001) and hope (F = 58.05, *p* < 0.001, small effect sizes), were expressed in the armed conflict crisis compared to the COVID-19 calamity. The only indicator which did not differ between the two measurements was the level of morale.

**Table 2:** General Linear Model – repeated measures, mean and standard deviation of coping mechanism (*N* = 593)

Coping mechanism	Variable	Scale	COVID-19		Armed conflict		F	Size effect ηp2
			**M**	**S.D**	**M**	**S.D**		
Distress	Danger	1–5	2.859	.767	2.498	.836	196.11***	.249
	Anxiety	1–5	2.535	.917	2.439	1.054	7.842**	.013
	Depression	1–5	2.203	.984	2.125	1.037	5.416*	.009
Resilience	IR	1–5	3.430	.728	3.478	.752	5.65**	.009
	CR	1–5	3.131	.895	3.316	.898	63.54***	.097
	SR	1–6	3.145	.891	3.707	.895	60.41***	.437
Subjective well-being	Well-being	1–6	3.938	.928	4.254	.957	110.26***	.157
	Hope	1–6	3.188	1.024	3.471	.978	58.05***	.089
	Morale	1–5	3.330	.951	3.320	.965	.050	.000

**p < .01, ***p < .001

*IR * Individual resilience, *CR*  Community resilience, *NR*  National resilience, *WB*   Well-being, M  Mean, S.D Standard deviation

To examine our second hypothesis, we used Pearson correlations among the seven coping mechanisms (Table 3). Results supported our hypothesis indicating similar patterns of correlations among the study variables across the two measurements: (a) The two-distress mechanism (distress symptoms and sense of danger) significantly and positively correlated with each other. (b) The three types of resilience (individual, community, and national) correlated significantly and positively with each other, and with the well-being mechanism. (c) The distress mechanism negatively correlated with both resilience and well-being measures.

**Table 3:** Pearson correlations among the coping mechanism across the two measurements

**Variable**		**2**	**3**	**4**	**5**	**6**	**7**	**8**	**9 Morale**
1. Danger	T1	.503***	.458***	-.354***	.155***	-.205***	-.460***	-.287***	-.435***
	T2	.623***	.603***	-.364***	-.211***	-.285***	-.459***	-.292***	-.501***
2. Anxiety	T1		.777***	-.466***	-.183***	-.182***	-.549***	.314 ***	-.623***
	T2		.796***	-.425***	-.194***	-.185***	-.529***	-.244***	-.662***
3. Depression	T1			-.443***	-.275***	-.278***	-.654***	-.413***	-.693**
	T2			-.459***	-.265***	-.281***	-.663***	-.387***	-.698***
4. IR	T1				.285***	.121**	.446***	.334***	.498***
	T2				.299***	.254***	.562	.423***	.512***
5. CR	T1					.404***	.378***	.350**	.337***
	T2					.448***	.380***	.395***	.280***
6. NR	T1						.290***	.488***	.324***
	T2						.350***	.558***	.311***
7. Wellbeing	T1							.460***	.682***
	T2							.357***	.661***
8. Hope	T1								.497***
	T2								.453***

*p < .05, **p < .01, ***p < .001, T1 = COVID-19, T2 = armed conflict

**Fig. 1:**
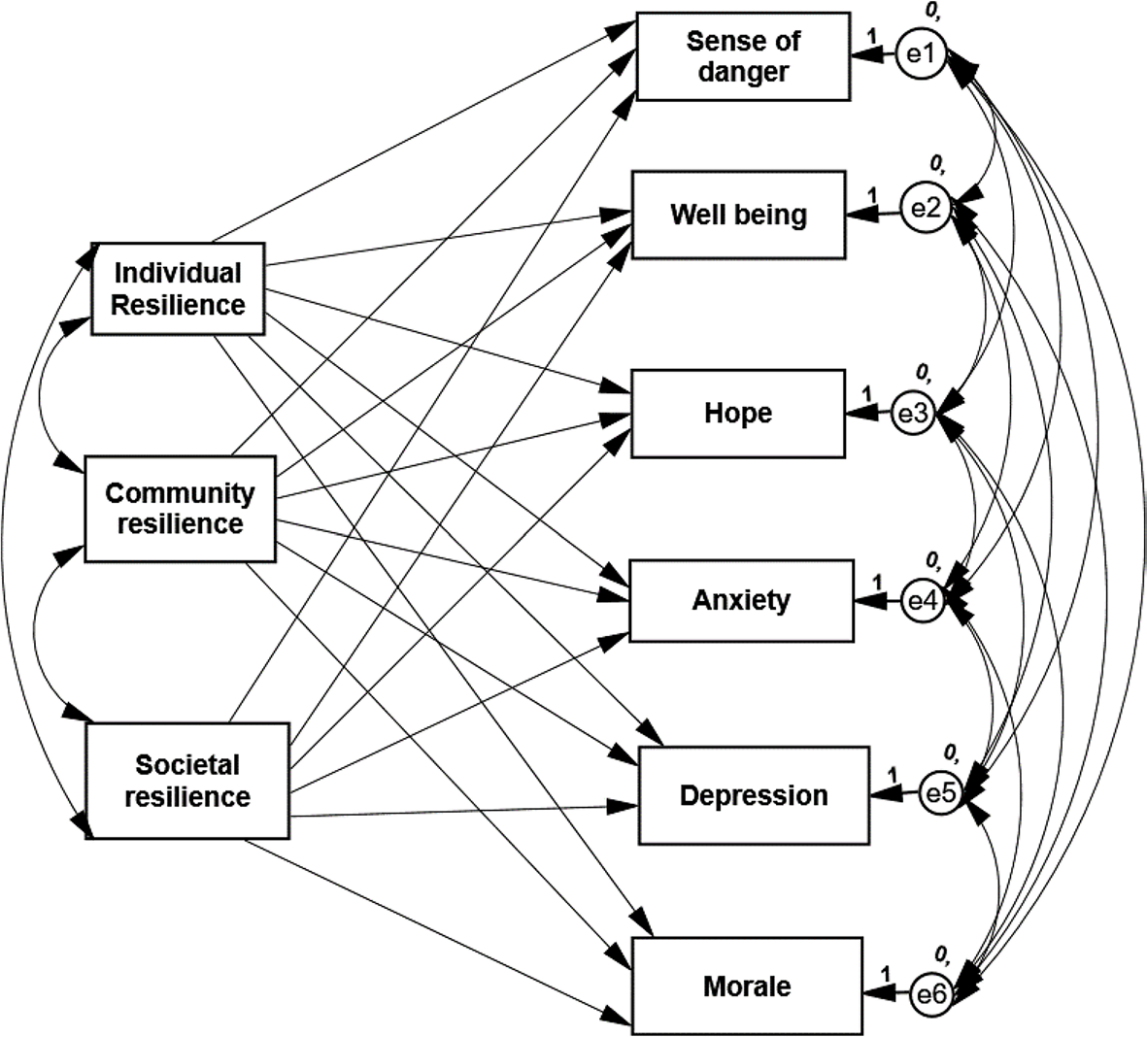
Example model of path analysis—Three types of resilience predicting six coping during COVID-19 and armed conflict

Two similar path analyses [46] were conducted on the data collected during the two adversities—COVID-19 and the armed conflict, to examine the third hypothesis on predicting the seven psychological coping mechanisms by the three types of resilience. Maximum likelihood estimates and a saturated model were employed, as we did not find any studies that supported an alternative model. It is important to note that in a saturated model, there is no need to examine the model fit, as the default and saturated models are the same [47] (Table 4 and Fig. 1). The results indicated the following: (a) Individual resilience predicted significantly and negatively the sense of danger, anxiety, and depression symptoms in each of the two adversities: The higher the IR, the lower the sense of danger, anxiety, and depressive symptoms. (b) Individual resilience predicted significantly and positively well-being, hope, and morale in both adversities: The higher the IR, the higher well-being, hope, and morale. (c) Societal resilience (SR) significantly predicted the investigated coping mechanism in the same direction, although its prediction of anxiety was not significant. (d) Less consistent results were obtained for community resilience. CR predicted positive and significant well-being and hope in both adversities but predicted positive morale only in the COVID-19 context. CR did not predict significantly the three negative mechanisms of adjustment: the sense of danger, anxiety, and depression.

**Table 4:** Standardized estimated path analysis of three types of resilience predicting six coping mechanisms during COVID-19 and armed conflict (*N* = 539)

Predictor	Predicted	COVID Estimate	Armed Conflict Estimate
Individual resilience	Sense of danger	-.336***	-.300***
	Well-being	.389***	.472***
	Hope	.252***	.278***
	Anxiety	-.450***	-.394***
	Depression	-.396***	-.399***
	Morale	.436***	.449***
Community resilience	Sense of danger	.008	-.060
	Well-being	.202***	.170***
	Hope	.111**	.117***
	Anxiety	-.003	-.048
	Depression	-.082	-.081
	Morale	.123***	.072
Societal resilience	Sense of danger	-.167***	-.181***
	Well-being	.161***	.154***
	Hope	.412***	.435***
	Anxiety	-.126***	-.064
	Depression	-.179***	-.143***
	Morale	.221***	.165***
Explained Variance (R^2^)	Sense of danger	.15	.17
	Well-being	.30	.38
	Hope	.32	.41
	Anxiety	.23	.19
	Depression	.25	.24
	Morale	.33	.30

**p < .01, ***p < .001

To ensure that each construct measures the variables it is planned to assess, the discriminant validity of the three resilience measures was assessed. As can be seen in Table 5, discriminant validity was found concerning all three measures.

**Table 5:** Checking for the discriminant validity of the three resilience constructs

Item	Number	Discriminate Validity
Average variance extracted NR	0.46	
Average variance extracted CR	0.61	
Average variance extracted IR	0.55	
Variance extracted between NR & CR	0.56	Variance extracted > correlation square, hence discriminant validity established
Correlation between NR & CR	0.38	
Correlation square	0.14	
Variance extracted between NR & IR	0.50	Variance extracted > correlation square, hence discriminant validity established
Correlation between NR & IR	0.11	
Correlation square	0.01	
Variance extracted between CR & IR	0.58	Variance extracted > correlation square, hence discriminant validity established
Correlation between CR & IR	0.27	
Correlation square	0.07	

## Discussion

The uniqueness of the current study lies in examining responses of the same sample of the population, during two different types of adversities. The levels of distress, resilience, and well-being were assessed during the peak phase of the COVID-19 pandemic and were re-assessed seven months later, during an armed conflict. Our main aim was to identify similarities and differences in coping indicators mechanism among this sample, in dealing with these two different adversities that co-existed within a relatively short period.

The review of the existing literature regarding coping with different types of adversities indicates that this issue has received little research coverage. The present study is designed to add to the existing knowledge on this subject, to improve the ability of the professionals to provide help, as well as to deal with various types of adversities and types of disasters (e.g., [48, 49].

Our main hypothesis that the participants sensed a greater difficulty in coping with COVID-19 compared to an armed conflict, was supported by most of the examined coping mechanisms. Responses during this pandemic were characterized by higher levels of sense of danger and distress symptoms, accompanied by lower levels of individual, community, and national resilience, well-being, and hope. The only exception to this rule was the level of morale that did not differ between the two examined adversities.

There is reason to believe that the greater difficulty of coping with the COVID-19 pandemic, which was found in this study, portrays the perceived difference in the characteristics of these adversities in the eyes of the investigated sample, rather than an actual greater threat posed by the pandemic compared to the armed conflict. These characteristics include (a) Familiarity with the threat. Israeli citizens are more familiar with repeated rounds of armed conflicts between Israel and the Gaza Strip, as they erupted several times in the past decade [50]. In contrast, the COVID-19 pandemic was an emerging threat, that occurred for the first time in recent years, which involves unknown immediate consequences and future impacts. (b) The duration of the adversity. Most armed conflicts in the region last for a relatively short duration, from a few days to a few weeks [51]. In contrast, the COVID-19 pandemic, at the time of measurement, had lasted for over a year, and its end is still not foreseen. (c) The number of casualties. The number of confirmed cases of the pandemic, as well as the overall mortality and morbidity, were much higher, compared with the regional armed conflicts, and future cases cannot as yet be predicted. (d) Consequently, the level of uncertainty due to the COVID-19 pandemic was much higher, compared with the armed conflicts in the Middle East [7].

A similar pattern of associations was found among the six coping mechanisms, across the two adversities. As expected, positive coping mechanisms were positively correlated with each other, distress mechanisms were positively correlated with each other, and these two groups of variables negatively correlated with each other. These associations were repeated in the two investigated adversities. These results suggest that the associations between distress, resilience, and well-being are stable, beyond the specific type of adversity [9, 10]. Our study suggests that those who found it difficult to cope with the COVID-19 crisis also found it difficult to cope with the armed conflict that followed and vice versa.

As previously identified, people who show high levels of stress and low resilience, for example, are likely to show similar responses across different adversities [25]. It is recommended that this conjecture be further researched by studies that will compare the coping mechanisms of individuals across different adversities and different cultures. These results support other studies that used a different method of analyzing repeated measures, such as Latent Growth Mixture Modeling (LGMM), tracking patterns of change over time [52–54]. These studies have indicated that most people who cope with adversity present a similarly low level of distress across time and types of adversity [23, 55]. However, these studies did not compare repeated measurements across varied adversities but concentrated on the same sample at different times of the same single adversity.

Resilience is vital for individuals and societies at large, regardless of the type of adversity, because all crises disrupt the social, cultural, and organizational systems and practices [56]. The importance of resilience for psychological adjustment during adversity is investigated in this study, by examining the extent to which the three kinds of resilience predict the investigated coping and the distress mechanism. The results clearly show that both IR and SR are strong and consistent predictors of coping and maintaining psychological adjustment in each of the two adversities examined in the current study. CR was found to be a less consistent predictor of efforts to cope with these risky circumstances.

### Limitations

Three main limitations, concerning the present study, should be noted: First, the study is based on self-report measures, and thus there may be a reporting bias. Second, it is a correlative study that does not allow for inference to conclude causalities. Third, the study sample is based on an online internet panel that does not necessarily represent a random sample of the entire population, most likely it does not include individuals without digital literacy.

## Conclusions

Our results show that psychological coping with different crises mainly reflects the perceived characteristics of each crisis, rather than its objective level of risk for the individual. People seem to regard unfamiliar, less predictable adversities, whose future development and implications are less well-known, as more complex to cope with, compared to better-known crises. Consequently, they often tend to underestimate the risks of a potential familiar adversity. These results further suggest that the associations between stress, resilience, and well-being mechanisms tend to be stable across two different adversities (the COVID pandemic and an armed conflict).

### Future research direction

As the commonalities and diversities in coping with different adversities have hardly been studied to date, it is recommended that additional research will be made, considering varied cultural contexts, types of societies, and different types of adversities. Furthermore, future research should investigate coping with adversities different from those examined in the present study, as well as in other cultures.

## Data Availability

The processed data that support the findings of this study are available from the corresponding author upon reasonable request. Requests to access the datasets should be directed to adini@tauex.tau.ac.il.
